# Exploring Acceptance of a Clinical Workflow Tool in the Swedish Prosthetics and Orthotics Sector: Qualitative Study

**DOI:** 10.2196/82584

**Published:** 2026-05-05

**Authors:** João Rafael Calixto, Nadia Davoody

**Affiliations:** 1 Health Informatics Centre, Department of Learning, Informatics, Management, and Ethics, Karolinska Institutet, Tomtebodavägen 18 A, Stockholm, S-17177, Sweden, 46 (0)8 524 864 86

**Keywords:** assistive devices, prosthetics and orthotics, user acceptance, clinical workflow, UTAUT, unified theory of acceptance and use of technology, thematic analysis

## Abstract

**Background:**

The global demand for assistive devices, such as prosthetics and orthotics, is increasing. A shortage of trained professionals contributes to suboptimal care. To improve clinical workflows, the Life Lounge Clinical Workflow (LLCW) has been developed. Understanding user acceptance is essential for ensuring its successful implementation.

**Objective:**

This study explored prosthetists and orthotists professionals’ perceptions and acceptance of LLCW, as well as the perceived benefits and challenges associated with its use.

**Methods:**

A postdemonstration mixed methods study was conducted using the unified theory of acceptance and use of technology framework, combining Likert-scale summaries with thematic analysis of open-text responses. The study included 18 prosthetists and orthotists professionals working at orthotic and prosthetic clinics across Sweden. After an interactive session about LLCW, feedback was collected via questionnaires. Thematic analysis was used to analyze the data.

**Results:**

Participants rated several acceptance-related constructs positively. Performance expectancy and facilitating conditions emerged as the most favorably discussed areas in the qualitative responses. Descriptive ratings showed high mean scores for motivation to use (4.61), management encouragement (4.56), ease of use (4.11), and willingness to use voluntarily (4.11). However, colleagues’ perceptions had a lower mean rating (2.72). Participants highlighted centralized data access, reduced administrative tasks, and improved clinical preparation as key benefits. At the same time, concerns were raised regarding data accuracy, questionnaire length, and the need for structured training before implementation.

**Conclusions:**

Participants reported generally positive experiences with LLCW, particularly regarding usability and performance. However, successful implementation requires integration into existing clinical workflows and attention to training and patient engagement. Addressing these elements can support broader adoption and contribute to digital transformation in prosthetics and orthotics care.

## Introduction

### Background

Assistive devices (ADs) are defined as “any item, piece of equipment, or product system, whether commercially acquired, modified, or customized, that is used to enhance, maintain, or improve the functional capabilities of individuals with disabilities.” [1]. Among these, mobility devices, particularly prosthetics and orthotics (P&O), are the most frequently needed [2], with global demand rising due to aging populations and the increased prevalence of disabling conditions [3,4].

Estimates of the global need vary, with some reports suggesting that up to 2.4 billion people may benefit from ADs by 2050 [2,3,5,6]. However, the ability to meet this growing demand is hampered by a shortage of trained professionals [3,5].

### The Role of Prosthetists and Orthotists

Prosthetists and orthotists professionals play a vital role in the comprehensive assessment of patients, considering physical, functional, psychosocial, and environmental factors, as well as in the design, fitting, and adjustment of ADs [6]. Yet, the number of trained professionals is insufficient to match the growing patient population. In Canada, for example, 37% of individuals report unmet needs for ADs [7]. In Sweden, where P&O training is offered only at Jönköping University, a national shortage is acknowledged, suggesting similar challenges in service delivery [8]. This shortage is associated with delayed care, extended rehabilitation timelines, and suboptimal patient outcomes [3,6].

Beyond workforce limitations, inefficiencies in the clinical workflow also affect care delivery. The process of prescribing a prosthetic device typically involves multiple professionals across 3 stages: physician referral, prosthetist assessment and fitting, and rehabilitation therapy [4]. However, the lack of standardized decision-making protocols often results in variation in device selection and unequal patient outcomes. For instance, in Sweden, regional guidelines differ, leading to inconsistent levels of care across clinics [9], further exacerbating inconsistencies in care.

### Workflow Inefficiencies

Another concern is the limited follow-up after device delivery. Patients often reconnect with their prosthetists only when problems arise, making it difficult to track long-term outcomes or ensure continuous use. This is critical, as discontinuation of AD is a common issue. Studies have found abandonment rates as high as 40% in some populations, with reasons including discomfort, poor training, and inadequate follow-up [10-12]. Discontinued use is not only associated with poorer health outcomes but also imposes additional economic strain due to the cost of unused or replacement devices [10-15].

Reimbursement models in the P&O field further compound these challenges. In many settings, including Sweden and the United States, reimbursement is based on a fee-for-service model that emphasizes device provision over patient outcomes [16]. This misalignment can lead to inefficiencies and insufficient incentives to support ongoing patient engagement. As health care systems move toward value-based models [17-19], there is an increasing demand for solutions that promote better outcomes while optimizing workflow.

### Digital Solutions: Life Lounge Clinical Workflow

One such solution is Life Lounge Clinical Workflow (LLCW), a digital platform developed by Ottobock to support prosthetists and orthotists professionals in managing patient data, monitoring progress, and enhancing interprofessional communication [20]. LLCW centralizes records, enables data transfer, and includes tools such as Smart Documentation and Custom Fabrication to streamline administrative processes and personalize care.

These features aim to reduce documentation burden, improve decision-making, and increase patient engagement, thereby addressing some of the key inefficiencies in current AD workflows.

LLCW, although not yet deployed in Sweden, has been used since 2023 in US clinics with positive feedback.

LLCW proposes to enhance the workflow by:

- Increasing patient involvement through previsit digital questionnaires
- Reducing clinician documentation via automated data capture
- Integrating all treatment steps into a unified digital platform
- Optimizing visit length by enabling clinicians to prepare in advance
- Improving long-term follow-up with routine patient-reported outcomes

These key changes aim to streamline the clinical process, promote patient-centered care, and improve overall efficiency.

### Technology Acceptance

Despite the benefits of LLCW, immediate acceptance by Swedish prosthetists and orthotists professionals is not guaranteed. Health care technology adoption can face resistance or unintended consequences if imposed without considering user concerns [21,22]. This study aims to optimize LLCW deployment by first understanding acceptance and addressing potential barriers.

Several frameworks assess technology acceptance, particularly in health care, where new tools impact workflows. Two widely used models are the technology acceptance model (TAM) [23] and the unified theory of acceptance and use of technology (UTAUT). TAM focuses on Perceived Usefulness and Perceived Ease of Use. While TAM is foundational, it has been criticized for oversimplifying the adoption process, leading to extended versions such as TAM2 and TAM3 [23]. On the other hand, UTAUT [24] emphasizes factors including performance expectancy (PE), effort expectancy (EE), social influence (SI), facilitating conditions (FC), and behavioral intention (BI), which provides a more comprehensive framework for understanding technology acceptance.

Although TAM and its extensions have been widely used, their limited ability in capturing the full complexity of acceptance [25-27] makes UTAUT a more complete framework to assess technology acceptance.

### Aim and Research Questions

The main aim of this study is to assess Swedish prosthetists and orthotists professionals’ acceptance of LLCW based on the UTAUT framework and to explore additional contextual factors that may support or hinder a future deployment in Sweden. To address this aim, the following research question guides the study: How do UTAUT-related factors and clinicians’ perceived benefits and shortcomings of LLCW shape their overall acceptance of the platform, and what elements should be prioritized to improve its suitability for future deployment in the Swedish P&O context?

## Methods

### Study Design

A qualitative, exploratory approach was adopted to investigate clinicians’ perceptions of LLCW. The study was situated within a constructivist-interpretivist paradigm [28], which assumes that clinicians’ experiences and acceptance of new technologies are shaped by individual interpretations and contextual factors. This paradigm aligns with capturing subjective perceptions rather than measuring predefined outcomes.

This method was selected as the research focuses on understanding individual experiences and acceptance, which is consistent with established qualitative practices in health care technology studies [29,30]. The study aimed to evaluate immediate acceptance and perceived usability following a demonstration session, combining descriptive Likert-scale summaries with thematic analysis of open-ended responses. This approach has previously proven effective in similar studies applying frameworks such as UTAUT to assess technology acceptance [24,31-34].

### Researcher Characteristics and Reflexivity

The primary researcher has an academic background in P&O and experience with technology-supported clinical workflows. These professionals’ experiences informed the formulation of the research aim and contributed to an initial expectation that digital platforms such as LLCW may support clinical efficiency and documentation quality. The researcher had no prior clinical relationship with the participating clinicians, which minimized direct power dynamics; however, shared professional terminology and familiarity with P&O practice may have influenced the interpretation of participant responses.

Throughout the study, reflexive attention was given to how these professional assumptions could shape data collection and analysis. Iterative discussions within the research team were used to monitor the potential impact of researcher perspectives on coding decisions and theme development. These measures were intended to strengthen transparency and support the transferability of findings by clearly acknowledging the interpretive position from which the analysis was conducted.

### Study Setting and Participants

The study was conducted at Ottobock Care Sweden, which in total operates 20 clinics across the country. Participants were recruited from these clinics; however, only prosthetists and orthotists professionals were included, as they are directly involved in patient care and their roles are most relevant to the use of LLCW. Clinic managers selected participants based on availability, reflecting a convenience sampling approach targeting 2‐5 clinicians per site. The inclusion criteria required participants to be actively engaged in patient care (regardless of years of experience) and to be a current employee of Ottobock Care Sweden.

Convenience sampling was chosen because it allowed the study to recruit clinicians without disrupting ongoing clinical operations and to access participants across geographically dispersed clinics. Decisions regarding whether further sampling was necessary were guided by the concept of thematic saturation at the overall dataset level. Saturation was monitored during early analysis to determine whether additional recruitment was likely to generate new themes.

### Data Collection

#### Overview

Due to the fact that LLCW is not yet deployed in Sweden, participants first attended a demo session where they were introduced to the platform and had the chance to explore its features. Demonstrations were delivered by members of the study team, independent of participants’ supervisors or routine clinical management. Participation was voluntary, and individual questionnaire responses were anonymized; clinic managers did not have access to individual answers. To reduce social desirability bias, participants were explicitly informed that their feedback would not influence performance evaluations or workplace standing.

To minimize disruption to clinical operations, the study used questionnaires rather than interviews or focus groups. This approach allowed participants to respond at their convenience, reduced time demands on clinical staff, and supported greater geographical reach. The instrument consisted of two integrated components: (1) open-ended questions designed to elicit qualitative feedback regarding benefits, shortcomings, and contextual factors, and (2) 6 Likert-scale items assessing perceived usability and acceptance in alignment with key UTAUT constructs. The questionnaire was administered digitally via Microsoft Forms, which also served as the platform for secure data storage.

Participating clinics were selected based on willingness and availability. Clinic managers were contacted via email with study details. Out of 20 clinics, 5 agreed to participate—located in Stockholm, Helsingborg, Kristianstad, Lund, and Malmö.

Data collection was conducted on-site at each clinic, except for Kristianstad, Lund, and Malmö, which were grouped at the Malmö location. Two sessions were held at the Stockholm clinic due to participant availability. Each session lasted 1.5 hours and followed a structured format. The introduction, lasting approximately 10 minutes, provided an overview of LLCW, a comparison with current workflows, and an explanation of the study objectives. Written consent was obtained during this time. The remaining 80 minutes were dedicated to hands-on interaction, during which clinicians, grouped by specialization, performed tasks such as testing intake forms and exploring other system functionalities.

Data were collected during March-April 2025. The procedures remained consistent across all clinics, and data collection included iterative monitoring of emerging patterns; however, no modification to the procedures was required.

In total, 18 prosthetists and orthotists professionals participated, and all completed the postdemo questionnaire, yielding a 100% response rate.

#### Questionnaire Design and Testing

The questionnaire was developed based on the UTAUT framework and included 6 sections: Demographics (5 items), PE (4 items), EE (4 items), SI (3 items), FCs (4 items), and BI (3 items). The full questionnaire is available in Multimedia Appendix 1.

To ensure clarity and validity, 2 rounds of pretesting were conducted. The first involved cognitive pretesting with 3 prosthetists and orthotists professionals (via debrief interviews), focusing on question clarity, interpretability, and completion time. The second consisted of an expert review by an Ottobock employee, emphasizing questionnaire completeness and technical functionality. Revisions were made based on feedback from both phases to ensure high response quality and user comprehension.

### Data Processing

All questionnaire responses in Microsoft Forms were reviewed for completeness, duplicate submissions, and formatting inconsistencies before coding began. Before analysis, each response was assigned a unique ID by the primary researcher to ensure full anonymization.

### Data Analysis

A 2-stage thematic analysis [35] was conducted using both deductive [36] and inductive [37] approaches, framed around the UTAUT model. In the deductive stage, responses were initially categorized according to the 5 UTAUT constructs. In the subsequent inductive stage, open-text responses within each UTAUT category were further analyzed to identify emerging subthemes using reflective thematic analysis [38]. Construct-specific coding was applied as follows: for PE, codes reflected perceived impact on clinical efficiency and patient care; for EE, codes addressed ease of learning, usability, and potential barriers; for SI, the analysis focused on the influence of colleagues, management, and organizational culture; for FC, coding covered availability of technical support, resources, and training; and for BI, codes captured participants’ intentions to use the system in future practice. Quantitative data from Likert-style responses were analyzed alongside qualitative findings. Relationships between constructs (eg, EE and BI) were explored. As all forms were fully completed, no missing data issues arose. PE and FCs were captured solely through open-ended qualitative responses, with no corresponding Likert-scale ratings. By triangulating quantitative and qualitative results, the study provides a comprehensive view of factors influencing LLCW acceptance and offers insight into how the platform can be better tailored for successful implementation.

### Techniques to Enhance Trustworthiness

To enhance credibility and dependability, the analysis followed a documented analytical workflow, including iterative coding, reflexive memo-writing, and repeated comparison between codes and raw data. Triangulation of qualitative themes with descriptive Likert-scale results strengthened the confirmability of interpretations. The reporting of this study followed the Standards for Reporting Qualitative Research (SRQR) guidelines (Checklist 1) to ensure methodological transparency, rigor, and completeness [39].

### Ethical Considerations

This study was carried out in Sweden. According to the Swedish Ethical Review Act (SFS 2003:460) [40] and the guidelines of the Swedish Ethical Review Authority [41], ethical approval was not required for this research, as it did not involve any sensitive personal data as defined under the European General Data Protection Regulation (EU 2016/679) [42]. Nevertheless, we acknowledge the importance of maintaining ethical standards and confirm that all relevant ethical principles were followed, in accordance with applicable legislation, the Declaration of Helsinki [43], and recognized research best practices. Prior to data collection, all participants received full information about the study’s purpose, scope, and voluntary nature. No incentives were offered, and participants were free to withdraw at any time without consequence. Written informed consent was obtained from all participants. Confidentiality and anonymity were maintained throughout the research: identifiable data were securely stored and accessible only to the research team. Results are reported in aggregate form to prevent identification. However, in clinics with only 2 participants, the risk of indirect identification remained despite standard deidentification. To mitigate this, additional steps were taken, such as grouping professional experience into broader intervals (eg, 10-year ranges), to strengthen anonymity without compromising data integrity.

## Results

### Overview

A total of 18 participants were recruited for this study. Questionnaire data were analyzed using a deductive thematic approach guided by the 5 constructs of the UTAUT. Participant responses were organized by these constructs to identify key patterns. Irrelevant statements were excluded to maintain focus. Each UTAUT construct is presented in the following sections, supported by direct quotations to illustrate core findings. Responses are referenced by participant ID.

### Participants Characteristics

Background information was collected to describe the sample, including gender, age, and professional experience (summarized in Table 1). The participant group was evenly split, with 9 male and 9 female respondents (n=18). Participants represented a wide age range. Male participants ranged from 24 to 55 years of age (mean 39 years; median 36 years). Female participants ranged from 28 to 35 years (mean and median 32 years). These figures reflect the age distributions within each subgroup and are presented solely as descriptive characteristics.

**Table 1.** Participant characteristics.

Characteristic	Male (n=9)	Female (n=9)
Age range (years)	24‐55	28‐35
Years of experience	0‐25	2‐15

Regarding professional experience, male participants reported 0‐25 years of experience (mean 12; median 11) and female participants reported 2‐15 years (mean and median 8). As with age, these values are descriptive and not used to infer subgroup differences.

Overall, the sample included both early-career and experienced professionals, providing a range of perspectives for evaluating the platform.

### User Acceptance

To assess key factors influencing acceptance of LLCW, 6 Likert-scale questions (rated from 0 to 5) were included in the survey. These questions aimed to capture perceptions related to the platform’s usefulness, usability, SI, and likelihood of adoption. The average scores for each factor are summarized in Table 2.

**Table 2.** Descriptive statistics for Likert-scale items (means and medians) for life lounge clinical workflow adoption factors.

	Willingness to use voluntarily (Q5)	Ease of use (Q10)	Colleagues’ perceptions (Q14)	Management encouragement (Q15)	Recommendation (Q22)	Use motivation (Q23)
ID 1	5	3	3	5	4	5
ID 2	4	4	2	5	5	5
ID 3	4	4	3	5	5	5
ID 4	3	4	2	4	4	4
ID 5	5	5	3	5	5	5
ID 6	4	4	3	5	5	5
ID 7	5	5	2	5	4	5
ID 8	4	4	2	4	3	5
ID 9	4	4	2	5	4	4
ID 10	3	4	3	4	3	4
ID 11	4	4	3	4	4	4
ID 12	3	4	2	4	4	4
ID 13	4	4	3	4	3	4
ID 14	5	4	4	5	4	5
ID 15	5	5	4	5	4	5
ID 16	5	4	3	5	4	5
ID 17	4	5	3	4	4	5
ID 18	3	3	2	4	3	4
Mean	4.11	4.11	2.72	4.56	4.00	4.61
Median	4	4	3	5	4	5

Table 2 shows strong positive perceptions in several areas. “Use Motivation” (Q23) had a mean score of 4.61, “Management Encouragement” (Q15) had a mean of 4.56, and both “Ease of Use” (Q10) and “Willingness to use Voluntarily” (Q5) were rated with mean scores of 4.11. “Recommendation” (Q22) with a mean score of 4.00 indicates a general openness toward adopting LLCW, though with slightly less enthusiasm than for the previously mentioned items.

In contrast, the relatively lower score for “Colleagues’ Perceptions” (Q14), a mean of 2.7, implies that peer influence may not be a strong motivating factor in this context. This might suggest that LLCW adoption is currently seen as more of an individual or management-driven initiative, rather than a socially reinforced norm.

### Key Feedback and Identified Themes

In addition to Likert ratings, written responses were analyzed and categorized by UTAUT construct. Emerging themes reflect both perceived benefits and potential concerns (Table 3).

**Table 3.** Main identified themes.

UTAUT^a^ construct	Themes
PE^b^	Performance Improvement and Workflow Efficiency Patient Involvement and Data Accuracy
EE^c^	Ease of Use and Intuitive Design Accessibility and Usability Concerns
SI^d^	Confidence in Colleague Adoption Resistance and Technological Comfort Influence of Personal Experience and Feature Gaps
FC^e^	Support and Training Needs Infrastructure and Technical Barriers Regulatory and Organizational Challenges
BI^f^	Adoption, Intent, and Confidence Platform Design and Usability Motivation and Contribution to Development

a UTAUT: unified theory of acceptance and use of technology.

b PE: performance expectancy.

c EE: effort expectancy.

d SI: social influence.

e FC: facilitating conditions.

f BI: behavioral intention.

### Performance Expectancy

#### Performance Improvement and Workflow Efficiency

Across all responses, participants expressed a strong belief that LLCW would improve their job performance. Clinicians consistently emphasized that the platform could streamline workflows, increase clinical efficiency, and enhance the quality of care by providing faster, more organized access to patient data.

A frequently cited benefit was the centralization and structured presentation of patient information, which was seen as reducing the risk of loss of data or miscommunication while enabling better preparation for patient visits.

*I think that LLCW makes our lives easier, because all information is on the same platform, so it is easier for us to manage all information that we have about the patient and which devices the patient uses, the scans that we have, etc*.

[ID 15]

Participants also noted that having prefilled patient data would allow them to dedicate more time to direct clinical care and less to administrative tasks, supporting the delivery of more individualized and effective treatment.

*Since a good part of the information we need from the patient can be obtained in advance… we can focus directly on the treatment…*

[ID 3]

*The patient fills in the information themselves and cannot be influenced by a clinician. More value-creating time for the patient during the visit.*

[ID 4]

The system’s potential to increase patient involvement was also seen as a contributor to improved outcomes and more tailored interventions. Patient participation, starting with intake forms and appointment booking, was viewed as a way to foster engagement and shared responsibility.

*I think it can impact positively since the patient gets involved since the beginning, starting with the questions they get in advance, but even managing their bookings…*

[ID 8]

Moreover, respondents highlighted that LLCW could reduce repetitive administrative tasks and shorten appointment durations, leading to more efficient clinical workflows:

*It definitely saves us time by skipping asking the same questions… so we can be more efficient during appointment time and maybe by reducing the time needed to collect data, we could have more patients on that day.*

[ID 10]

*It may facilitate the administrative tasks and make the duration of the visit shorter by automatically entering the data from the folders that the patient is going to fill out at home and saving it in the system.*

[ID 8]

These efficiencies were seen as likely to improve overall clinic capacity and service delivery.

#### Patient Involvement and Data Accuracy

While the platform’s ability to engage patients was viewed positively, some participants expressed concerns about patient participation in data entry. Clinicians pointed out that not all patients are likely to complete the digital forms thoroughly, which could limit the effectiveness of the platform. One respondent noted:

*The challenge in patient cases, where the patient isn’t that involved themselves…*

[ID 1]

A particular concern was raised regarding older patients or those less familiar with digital technology. Clinicians questioned whether these individuals would be able to complete forms accurately and independently, as emphasized by the following statement:

*I think the information I can get from LLWC is good, but I have a hard time seeing how older patients will be able to fill out so many questions…*

[ID 4]

Another clinician emphasized the importance of verifying patient-provided information to ensure that clinical decisions are based on accurate and reliable data:

*I still believe that you need to spend time ensuring that the information is correct and that the right information has been received…*

[ID 4]

### Effort Expectancy

#### Ease of Use and Intuitive Design

Participant feedback generally reflected a positive impression of LLCW’s usability, with many describing the platform as intuitive, well-organized, and easy to navigate. The system’s clean interface and logical structure were frequently cited as contributing factors to a positive user experience. The separation of documents, scans, and files within the system was particularly appreciated, with one participant noting the usefulness of being able to preview scans directly without needing to open a new window.

*Easy to understand, clean interface that makes it clear where we have to click in order to get where we want. Really liked that the patient info is divided on documents/scanning/others so it’s easier to get directly to what we want and not search around on the same page. So good that we can see a preview of the scanning without needing to open it somewhere else.*

[ID 3]

This organizational clarity was seen as facilitating efficient access to patient information, thereby streamlining clinical workflows.

Another commonly noted strength was the consolidation of multiple tools into a single platform. This integration was seen as a time-saving feature, eliminating the need to switch between different systems currently used at the clinics:

*I think that it will be easier for us CPOs because all functions and information are on the same platform, reducing time between the meeting with the patient and the delivery of the device.*

[ID 12]

#### Accessibility and Usability Concerns

Despite the generally positive reception, participants raised concerns about the accessibility of LLCW, particularly for users with limited experience using digital technologies. Several clinicians pointed out that while they found the system easy to use, others, especially older colleagues, might struggle:

*I think that it will be easier for those who use computers and new technologies, but those who don’t use them can find it difficult.*

[ID 9]

*Felt easy to understand, though older colleagues might struggle in the beginning.*

[ID 11]

In line with these observations, similar concerns were raised regarding patient accessibility, particularly for older individuals or those less digitally literate. Participants were unsure whether patients would be able to navigate the system independently, especially the intake form, which was described as long and potentially overwhelming:

*I have a lot of older patients, who I’m not sure if they will be able to use it by themselves, especially the intake form part, which is quite long.*

[ID 7]

Additionally, technical limitations of the mobile interface were mentioned as potential usability barriers for both clinicians and patients. One participant noted issues with text visibility on mobile devices:

*…I couldn’t see all the text under the questionnaire via phone. Incorrect interface.*

[ID 4]

### Social Influence

#### Confidence in Colleague Adoption

Most participants expressed confidence that their colleagues would respond positively to LLCW, especially in terms of its potential to improve workflow.

*I think it would be positive, and the workflow could be more effective, making it welcome.*

[ID 11]

Supervisors were also seen as likely to support the system’s implementation, particularly due to its capacity to enhance clinical outcomes. One clinician highlighted the platform’s ability to provide data-driven support for treatment decisions:

*Adding more power behind decision making – the clinician will have an answer on whether the solution picked for the patient is the one with the best outcome.*

[ID 1]

Others echoed this sentiment by linking LLCW’s functionality with improved daily operations and time efficiency:

*I think that facilitates the work and reduces the time spent with the patients, so I think they will like LLCW.*

[ID 11]

#### Resistance and Technological Comfort

Despite general optimism, some participants anticipated resistance from colleagues, particularly from those who are less comfortable with digital technologies.

*I think that depends on the colleagues. Some will think that it is easier with that platform, and those who are not used to new technologies may think that it is not so easy.*

[ID 2]

This highlights how varying levels of technological familiarity may influence initial acceptance. Resistance was viewed as a common and expected reaction to change, but participants believed it would diminish over time with proper exposure and demonstrated benefits:

*Like everything that is new, there will always be some resistance to it, but as long as the platform can allow us to do the work we do right now in a better way, in time it will be a 5.*

[ID 13]

#### Influence of Personal Experience and Feature Gaps

While acknowledging that peer opinions may play a role, participants emphasized the importance of forming their own judgments based on personal use. This suggests that individual engagement with the system may ultimately outweigh SI:

*Partially, good to have input from others, but it is important to try it out yourself and not rely only on others’ experience with the LLCW.*

[ID 12]

Finally, a few participants noted that certain missing features could impact how positively their colleagues perceive the system. These included practical functionalities such as inter-clinic messaging, customizable scheduling, and billing integration:

*I think that would be perceived as positive. But I also think that certain parts would be missing, such as messaging between clinics, the ability to change the color of bookings in the schedule, and billing options for customers.*

[ID 4]

### Facilitating Conditions

#### Support and Training Needs

A key theme that emerged from participant feedback was the importance of training and implementation support for ensuring effective use of LLCW. For instance, one respondent highlighted the need to integrate key functions from current systems, such as the check-in process, into LLCW to facilitate a seamless transition:

*Implementation, training, ensuring that key functions of current systems are available in LLCW, like the check-in system for components.*

[ID 4]

While training was seen as a necessary component for many, some participants, especially younger clinicians, felt that less support might be needed. A few respondents believed that a basic understanding of the system would be sufficient for initial use.

*No need for extra support, only ensuring that everybody knows the basics of how to use it from the start.*

[ID 7]

#### Infrastructure and Technical Barriers

The infrastructure and technical readiness of the workplace were identified as important factors influencing the success of LLCW adoption. While some participants believed that their current infrastructure could support LLCW “to some degree,” concerns about IT support responsiveness were raised. One participant noted:

*…Currently, when we have [IT] problems, it’s hard to get support, so not sure if it will be different with Life Lounge.*

[ID 5]

Another respondent echoed this concern:

*IT problems happen from time to time now, so ensuring a faster response in case we use Life Lounge would be great.*

[ID 16]

Additionally, some participants suggested that IT support staff may need additional training to handle common problems more effectively:

*Support might need training [to address] common problems so they can help us fast.*

[ID 7]

This feedback highlights that, beyond infrastructure readiness, the technical competence of support staff is crucial for smooth system adoption.

#### Regulatory and Organizational Challenges

Participants also identified regulatory and organizational barriers as potential obstacles to implementing LLCW, particularly in different regions. One respondent raised concerns about legal and administrative issues that could delay the process:

*…In Sweden, it is difficult to add new platforms because they depend on the region for making that decision and because of the security of patient information…*

[ID 2]

Moreover, participants raised concerns about the ability of LLCW to accommodate the diverse workflows of clinics, especially when working with external health care providers.

*I think the hard part could be that different clinics have different treatment forms, connected to different hospitals…; the challenge could be incorporating a new workflow to the personnel/partners we work with.*

[ID 1]

#### User Manuals and Internal Expertise

In terms of user manuals, opinions varied depending on the participants’ age and experience level. Older participants were more likely to recommend the inclusion of a searchable help feature within the platform to support users:

*I think the manual could be implemented in the search function in the program (chat) to search for facts/tips and tricks in help text describing the function of different parts in LLCW.*

[ID 10]

Others suggested a combination of digital resources and internal expertise:

*…It would be nice to have 2/3 people on the clinic to be ‘experts’ on the system to which all others can turn to if the manual can’t help.*

[ID 3]

On the other hand, younger participants were less inclined to see a user manual as essential. Instead, they emphasized the importance of hands-on experience and initial training:

*No need, it will be easy to know how to use all functions with practice.*

[ID 5]

*Just ensuring that how-to-use sessions are provided in the beginning to teach us the basics.*

[ID 15]

### Behavioral Intention

#### Adoption Intent and Confidence

Participants exhibited a strong intention to adopt LLCW once it is implemented in their clinics, driven by a clear sense of optimism regarding the system’s ability to improve efficiency and provide better access to patient information. Several respondents expressed confidence in the platform’s value, especially in enhancing clinical processes and supporting patient care.

*…I think it could be a strong tool in the clinic…*

[ID 11]

The ease of access to patient data was frequently cited as a key benefit of the system, with several participants noting how it would streamline their daily workflows.

*I will use it all the time because it is easy to see the patients’ information…*

[ID 2]

Despite the positive feedback, some participants also acknowledged the challenges associated with the initial implementation of any new system. One respondent offered a more balanced perspective, noting:

*…If we implement a new system, we will, of course, encounter problems, but with time, I think we can overcome themy…*

[ID 4]

#### Platform Design and Usability

The platform design and user interface were particularly appreciated by several participants. Many emphasized that the clean, user-friendly design made the platform easy to navigate, which contributed to their confidence in using it.

*…I really like the interface and the idea behind it!*

[ID 3]

The centralized organization of information was also highlighted as an advantage. Participants appreciated how it allowed them to find everything they needed in one place, eliminating the time spent searching across multiple platforms.

*I think the platform facilitates our work, because it is easy to use and all the information is in the same place.*

[ID 12]

Additionally, many participants saw room for future development, expressing a willingness to see LLCW grow and evolve over time.

*I think it has strong potential, and of course, should be improved over time.*

[ID 1]

Moreover, the platform’s design was appreciated for being tailored to the specific needs of users, with one participant commenting:

*…It seems to have been developed to answer our needs.*

[ID 17]

#### Motivation and Contribution to Development

Motivation to adopt LLCW was also tied to the opportunity to contribute to the ongoing development of the platform. Some participants expressed a strong desire to be involved in future updates and to see their feedback integrated into the system.

*Also, even more motivated to see how we can improve it after starting to use it.*

[ID 3]

Another expressed interest in conducting broader implementation testing:

*I would like to conduct a larger test to see what possibilities exist for implementation.*

[ID 4]

Interestingly, the opportunity to have their voices heard and actively shape the system’s development seemed particularly significant to some participants.

*It will make us more effective. Also, like that, our opinions are being considered before, so there is the possibility to, at some point, work on the improvements we suggested.*

[ID 17]

### Areas for Improvement in LLCW

Based on feedback from participants, several areas for improvement in LLCW were identified. While the general sentiment towards the platform’s potential was positive, there are clear opportunities for enhancing its functionality and usability.

### Improving Mobile Usability

One of the pressing areas for improvement identified by participants was the usability of LLCW on mobile devices. Respondents mentioned that the platform’s layout and functionality on smartphones were not optimized, leading to issues like form resets when editing responses.

*It was easy to learn, I found it clear where I could find the information I needed. However, I couldn’t see all the text under the questionnaire via phone. Incorrect interface.*

[ID 4]

This was particularly frustrating for older patients, who might struggle to navigate the mobile interface.

*Yes, i think that the questionary can be difficult at answer if you are old, and 50% of our patients they are really old, maybe the time that we take to fill in the questionary also, it is a lot of questions that maybe we could scape that are not so important to our patients and devices and that could reduce the time.*

[ID 2]

These issues indicate that mobile optimization should be a priority in future updates. To make the platform more accessible, especially for older patients and those with limited technical skills, adjustments need to be made to ensure smoother navigation and fewer disruptions when completing forms.

### Integrating More Clinical Data Fields

Another gap identified in LLCW was the lack of clinical data fields within patient profiles. Participants noted that the platform currently does not capture crucial clinical information, such as strength scales, test results, or other relevant health data that clinicians routinely use to monitor and compare patient progress.

*I Would appreciate though to have a field on the patients’ information regarding our clinical data…*

[ID 3]

The absence of customizable clinical fields limits the platform’s ability to fully support health care workflows, as clinicians often rely on detailed patient histories to make informed decisions.

### Streamlining Data Entry and Reducing Patient Fatigue

The length and complexity of the questionnaires were flagged as another area for improvement, particularly for older patients, who represent a substantial segment of those treated. Several participants pointed out that lengthy forms can lead to patient fatigue, causing some individuals to rush through responses or abandon the process altogether. To improve this aspect, there is a strong call for simplifying the questionnaires and reducing their length, as participants recommended multiple times during demo sessions.

*Yes, i think that the questionary can be difficult at answer if you are old, 50% of our patients are really old, maybe the time that we take to fill in the questionary also, it is a lot of questions that maybe we could scape that are not so important to our patients and devices and that could reduce the time*.

[ID 2]

Additionally, during demo sessions, participants recommended introducing adaptive questioning—where the number of questions changes based on the patient’s responses—could make the process more efficient and less overwhelming.

### Enhancing Clinician Support and AI Integration

Several participants emphasized the need for improved support tools for clinicians within the platform. A suggestion was made to incorporate a forum or chat function, which would allow clinicians to access real-time support for troubleshooting or specific queries. As one respondent noted:

*We need a forum for questions and a chat function*.

[ID 1]

In addition to improving support, there were calls to integrate AI tools into LLCW to assist clinicians, especially when dealing with rare or complex cases. AI-driven assistance could help clinicians by providing real-time decision support, making it easier for them to access relevant information, identify patterns, and make more informed decisions.

By incorporating AI and enhanced support systems, LLCW could provide clinicians with more advanced tools, enabling them to handle challenging cases with greater efficiency and confidence.

*AI could be implemented as a way to support clinicians in those special cases (rare patient situations).*

[ID 1]

## Discussion

### Principal Results

This study examined acceptance of LLCW among Swedish prosthetists and orthotists professionals using the UTAUT framework. Overall, participants expressed strong BI to adopt the platform, primarily driven by high PE and EE. LLCW was perceived as intuitive and capable of improving workflow efficiency and patient care. However, concerns were raised regarding patient digital engagement, data accuracy, training needs, and long-term technical support, highlighting important considerations for successful implementation. Participants generally expressed positive acceptance of LLCW, highlighting its potential to improve job efficiency, enhance patient care, and simplify routine tasks. However, concerns about patient participation were raised, which need to be addressed for smoother implementation. Qualitative feedback on PE showed alignment between the platform’s expected benefits and users’ perceptions. Participants’ open-ended responses consistently described PE-related aspects most positively, suggesting perceived support for LLCW’s impact on efficiency and effectiveness.

Descriptive ratings indicated consistently positive perceptions of the platform, with high mean scores for motivation to use (4.61), management encouragement (4.56), willingness to use voluntarily (4.11), ease of use (4.11), and likelihood of recommending LLCW (4.00). These results reflect generally favorable user impressions and perceived value of the platform for supporting workflow and future engagement.

### Interpretation and Comparison With Existing Literature

In line with our objective to assess factors influencing acceptance of LLCW, findings indicate that while the platform was perceived as intuitive and valuable, workflow integration may present important challenges. The introduction of LLCW could require workflow adjustments, which may pose a significant barrier to its acceptance [44-46]. While the platform aims to improve workflows, such changes may inadvertently increase errors and clinician workload [47]. Concerns about patient participation, especially in submitting data independently, could complicate this. If patients feel unsure about or struggle to complete data submissions before in-person visits, they may delay or avoid the process, negating efficiency gains and increasing clinician workload. To address this, preimplementation efforts should assess patient behavior, like response rates to current postdelivery surveys, and develop strategies to promote active patient engagement in their new roles.

Although patient-reported data offers valuable insights, its quality may vary. The subjectivity involved in interpreting questions could compromise data accuracy [48], leading to potential treatment errors if clinicians rely too heavily on it. To optimize the value of patient-reported data, LLCW should implement mechanisms for verifying its accuracy. Automated approaches for data verification, as explored in existing literature [49], could be incorporated into the system to ensure data reliability and maximize its potential benefits. These findings suggest that although LLCW shows promise for improving efficiency and clinical preparation, successful implementation will depend on addressing workflow adaptation and ensuring data accuracy.

Participants’ positive outlook on PE aligns with previous literature, which consistently supports the significance of PE in the acceptance of health care technology. Studies by Ljubičić et al [24], De Mesa et al [31], and Klappe et al [32] identify PE as a key adoption driver within the UTAUT framework. The benefits of LLCW align closely with this construct, particularly in its ability to reduce documentation burdens and increase patient involvement. Since documentation is often regarded as a time-consuming task by clinicians [50], tools like LLCW that streamline such processes are well-received.

Participants generally had positive impressions of LLCW’s EE, with a mean of 4.11, reflecting its user-friendly design and aligning with earlier findings that EE significantly affects technology adoption [51]. However, concerns were raised about the ability of older patients, a significant portion of the caseload, to engage with the platform. Literature supports this, as older adults often have lower digital literacy in health apps [52,53]. This could hinder the expected benefits of LLCW in clinical practice and impact long-term acceptance. Since this study didn’t focus on patients, it is not possible to make definitive claims about their expected reaction; however, since older patients are known to have lower confidence and self-perceived tech skills [53], a patient introduction strategy similar to the one used with clinicians is essential to ensure proper support and reduce potential digital barriers.

Additionally, participants expressed concerns about the ability of their older colleagues to navigate LLCW, especially those who might have less comfort with technology. This was particularly voiced by older participants themselves, highlighting how age can influence perceptions of digital competence. This concern aligns with findings in the literature [54,55], but in this case, it was framed as an issue for colleagues rather than a personal one. Regardless, these concerns underline the importance of offering thorough training [33,51,56] to all health care providers, regardless of age (even though a trend among younger participants seeing training as unnecessary has been identified) or digital proficiency.

When it comes to SI, this construct seemed to be the least emphasized by participants in their feedback about LLCW, mirroring previous research where SI was found to be the weakest predictor of technology acceptance [34]. As shown in Table 2, SI received the lowest mean score of 2.72, which, while not negative, suggests it played a relatively neutral role in shaping participants’ intentions to adopt LLCW in the future. This indicates that the influence of peers, supervisors, or colleagues may not be a significant driving factor in their decision-making process. Participants reported strong (expected) managerial support for the adoption of LLCW, reinforced by expectations of operational gains. Although peer influence appeared limited in the previously mentioned findings, managerial endorsement seemed to play a greater role in shaping acceptance decisions. Despite assurances of anonymity, this discrepancy may reflect organizational pressure. The lack of expressed concerns about peer opinion may also indicate, however, confidence in the tool rather than disregard for social dynamics. If negative feedback were anticipated, SI might have played a larger role.

When it comes to FC, participants generally felt that their workplace infrastructure could support LLCW’s implementation, which might justify the expected acceptance, as strong infrastructure has been positively associated with technology acceptance [24,34,57]. However, concerns about existing IT support, such as slow response times and inefficiency, were raised. These issues could impact the platform’s long-term acceptance, as effective IT support is crucial for a smooth rollout [58,59]. If LLCW addresses these support issues, it could positively influence perceptions and adoption.

If LLCW fails to meet expectations, professionals may reassess their views, leading to frustration and reduced trust, which could hinder adoption [60,61]. Therefore, it’s important for LLCW to deliver on its promises to maintain positive engagement and long-term acceptance. Setting up strategies to follow up on this specific factor is then extremely important to consider postimplementation.

Regarding BI, participants showed overwhelmingly positive attitudes towards LLCW, with a strong inclination to adopt and recommend the system. The primary motivator for this was the expected efficiency improvements, which participants felt would streamline workflows and enhance clinical operations, making them eager to embrace the platform. This aligns with broader research showing that expected performance improvements are central to fostering adoption intent [24,31,32].

Participants also reported a sense of ease in using LLCW, with its user-centered design contributing to their confidence in its effectiveness and usability, key factors for overall satisfaction [51,62]. Many expressed satisfaction with having their opinions considered during the predeployment phase, fostering a sense of involvement and ownership. This approach sparked curiosity about seeing their feedback reflected in the platform’s development, and some participants expressed a desire to contribute to future improvements [63,64].

The appreciation for being consulted aligns with previous research, showing that involving stakeholders in the design and planning stages enhances technology acceptance and satisfaction in health care [24,31,32].

Such stakeholder engagement aligns with best practices in health care digitalization, where involving clinicians early in the design process has been shown to increase satisfaction and acceptance [21,22,62,64]. When users feel their input is valued and integrated, they are more likely to support the technology’s implementation [65,66]. While appreciation for this inclusive approach was voiced most explicitly by female participants, there is no clear evidence that gender itself influenced acceptance. Rather, the findings suggest that meaningful user involvement broadly contributes to positive adoption attitudes.

### Implications and Conclusions

Overall, the study’s findings reinforce existing research: PE and EE are the most positively rated constructs, while SI tends to be less influential. Strong FC and a user-inclusive design further bolster BI. LLCW’s perceived ability to improve efficiency, its intuitive interface, and stakeholder engagement all contributed to the favorable reception among health care professionals.

### Limitations

This study evaluated the acceptance of LLCW among Swedish prosthetists and orthotists professionals by applying the constructs of UTAUT. Nevertheless, certain limitations may have influenced the results. While the primary objective, accurately capturing participants’ views on LLCW, was achieved, alternative methods of data collection could have yielded richer qualitative data. For instance, focus groups, known for fostering collaborative discussion and the emergence of new ideas, might have provided deeper insight. Likewise, in-depth interviews could have enabled more probing, allowing researchers to explore individual perspectives in greater detail [67]. These methods were not viable due to operational limitations. Another limitation is the challenge of achieving participant saturation at the clinic level. In qualitative research, saturation refers to the point at which no new information or themes emerge [68]. While saturation appeared to be reached across the broader participant sample, the inclusion of clinics with very few participants (eg, n=2) may have hindered the discovery of additional relevant insights. A larger sample in these clinics could have strengthened the study’s comprehensiveness. The study also faced limitations in achieving demographic balance, particularly regarding variables such as age, which are known to influence UTAUT constructs. Since participants were selected based on availability rather than stratified sampling, the resulting demographic imbalances, such as age distribution, could have affected the results. Moreover, the brief nature of the LLCW demonstration may have limited participants’ ability to form well-rounded opinions. The session’s short duration, combined with the fact that participants were paired during the demo due to operational constraints, may have affected the quality and independence of the feedback collected. A more in-depth or individualized demo experience might have produced more nuanced responses.

It is also important to consider the potential influence of social desirability bias. Despite generally positive feedback, participants may have modified their responses to appear favorable to supervisors or colleagues [69]. Since LLCW was developed internally by Ottobock, there may have been an implicit pressure to evaluate it positively. Finally, while participants came from multiple clinic locations, no comparative analysis was conducted to examine potential differences in acceptance across these sites. Such an analysis could have helped identify the most suitable clinic for piloting LLCW and inform a more effective implementation strategy across Sweden.

### Conclusions

This study evaluated the factors influencing prosthetists and orthotists professionals’ acceptance of the LLCW platform. While overall feedback was positive, particularly regarding its usability and potential to enhance performance, participants emphasized that full acceptance depends on future improvements. Key among these is the integration of decision support systems tools, and strategies to support patient engagement.

Given the study’s small convenience sample and immediate postdemonstration design, these findings should be interpreted as preliminary insights. Future research with larger, more diverse samples (including secondary users such as patients) will be essential to guide broader implementation and inform strategies for long-term adoption across Ottobock Care Sweden clinics.

## Supplementary material

10.2196/82584 Multimedia Appendix 1 Questionnaire used in the study, including demographic items and adoption-related questions.

10.2196/82584 Checklist 1 SRQR checklist.

## Abbreviations

AD: assistive device
BI: behavioral intention
EE: effort expectancy
FC: facilitating condition
LLCW: life lounge clinical workflow
P&O: prosthetics and orthotics
PE: performance expectancy
SI: social influence
SRQR: Standards for Reporting Qualitative Research
TAM: technology acceptance model
UTAUT: unified theory of acceptance and use of technology
