# Supplementary material for: Exploring Acceptance of a Clinical Workflow Tool in the Swedish Prosthetics and Orthotics Sector: Qualitative Study
Source: JMIR Form Res. 2026 May 5;10:e82584. doi: 10.2196/82584 (PMC13143195; doi:10.2196/82584)
Supplement: Multimedia Appendix 1 [file formative-v10-e82584-s001.pdf]

Appendix 1

Full questionnaire used in the study, including demographic items and adoption-related questions covering Performance Expectancy, Effort Expectancy, Social Influence, Facilitating Conditions, and Behavioral Intention. The questionnaire includes both Likert-scale and open-ended questions.

**Question Number**	**Type**	**Question**
1	DEM	How old are you?
2	DEM	What is your gender?
3	DEM	How many years of clinical experience do you have?
4	DEM	What is your area of clinical specialization?
5	DEM	After your experience using LLCW on a scale from 0 to 5, how willing are you to voluntarily use it in your practice? (0 = Not at all willing, 5 = Very willing)
6	PE	How do you think using Life Lounge Clinical Workflow (LLCW) could affect your ability to manage patient information, and why?
7	PE	How do you think LLCW might influence the quality of patient care you provide, and why?
8	PE	How do you think LLCW could impact the involvement of patients in their treatment plans, and why?
9	PE	How do you think having access to more patient information in advance might affect administrative tasks and the duration of visits, and why?
10	EE	How easy do you think it will be to learn to use Life Lounge Clinical Workflow (0–5)? Could you elaborate on your answer?<br>• 0 – Very difficult<br>• 1 – Difficult<br>• 2 – Somewhat difficult<br>• 3 – Neutral<br>• 4 – Easy<br>• 5 – Very easy
11	EE	Did you face any challenges using Life Lounge Clinical Workflow? If so, which ones? If not, can you foresee any challenges for other indirect users, such as patients?

12	EE	How do you think Life Lounge Clinical Workflow's user interface will impact your ability to perform tasks, and why?
13	EE	Do you think the usability of Life Lounge Clinical Workflow could be improved, and why?
14	SI	How do you think your colleagues will perceive Life Lounge Clinical Workflow (0–5)? Could you elaborate on your answer?<br>• 0 – Very negatively<br>• 1 – Negatively<br>• 2 – Somewhat negatively<br>• 3 – Neutral<br>• 4 – Positively<br>• 5 – Very positively
15	SI	To what extent do you think your supervisors will encourage the use of Life Lounge Clinical Workflow in your work (0–5)? Could you elaborate on your answer?<br>• 0 – Not at all<br>• 1 – Slightly<br>• 2 – Somewhat<br>• 3 – Neutral<br>• 4 – Moderately<br>• 5 – Strongly
16	SI	How would your colleagues' use of LLCW influence your own decision to use it, and why?
17	FC	What additional support or resources would make it easier for you to use Life Lounge Clinical Workflow effectively?
18	FC	How do you think the existing infrastructure and resources at your workplace will support the use of Life Lounge Clinical Workflow, and why?
19	FC	What barriers (technical, organizational, or personal) do you foresee that might make it difficult to use Life Lounge Clinical Workflow?
20	FC	Do you think having access to a user manual would facilitate your usage of Life Lounge Clinical Workflow in your workflow? If so, for which features would you require it the most?
21	BI	How likely do you see yourself using Life Lounge Clinical Workflow once it becomes available in your clinic, and why?
22	BI	How likely are you to recommend LLCW to others once you start using it (0–5)? Could you elaborate on your answer?

		• 0 – Not at all<br>• 1 – Slightly<br>• 2 – Somewhat likely<br>• 3 – Neutral<br>• 4 – Likely<br>• 5 – Extremely likely
23	BI	How motivated are you to incorporate LLCW into your work processes in the future (0–5)? Could you elaborate on your answer?<br>• 0 – Not at all<br>• 1 – Slightly<br>• 2 – Somewhat motivated<br>• 3 – Neutral<br>• 4 – Motivated<br>• 5 – Highly motivated
